# BcWRKY22 Activates *BcCAT2* to Enhance Catalase (CAT) Activity and Reduce Hydrogen Peroxide (H_2_O_2_) Accumulation, Promoting Thermotolerance in Non-Heading Chinese Cabbage (*Brassica campestris* ssp*. chinensis*)

**DOI:** 10.3390/antiox12091710

**Published:** 2023-09-01

**Authors:** Haiyan Wang, Zhanyuan Gao, Xiaoshan Chen, Entong Li, Ying Li, Changwei Zhang, Xilin Hou

**Affiliations:** 1 State Key Laboratory of Crop Genetics & Germplasm Enhancement and Utilization, Key Laboratory of Biology and Genetic Improvement of Horticultural Crops (East China), Engineering Research Center of Germplasm Enhancement and Utilization of Horticultural Crops, Nanjing Agricultural University, Nanjing 210095, China; 2021104053@stu.njau.edu.cn (H.W.); 2021204025@stu.njau.edu.cn (Z.G.); 2019204020@njau.edu.cn (X.C.); 2021204020@stu.njau.edu.cn (E.L.); yingli@njau.edu.cn (Y.L.); 2 Nanjing Suman Plasma Engineering Research Institute Co., Ltd., Nanjing 211162, China

**Keywords:** non-heading Chinese cabbage, BcWRKY22, thermotolerance, *BcCAT2*, catalase (CAT), H_2_O_2_

## Abstract

WRKY transcription factors (TFs) participate in plant defense mechanisms against biological and abiotic stresses. However, their regulatory role in heat resistance is still unclear in non-heading Chinese cabbage. Here, we identified the WRKY-IIe gene *BcWRKY22*(*BraC09g001080.1*), which is activated under high temperatures and plays an active role in regulating thermal stability, through transcriptome analysis. We further discovered that the BcWRKY22 protein is located in the nucleus and demonstrates transactivation activity in both the yeast and plant. Additionally, our studies showed that the transient overexpression of *BcWRKY22* in non-heading Chinese cabbage activates the expression of catalase 2 (*BcCAT2*), enhances CAT enzyme activity, and reduces Hydrogen Peroxide (H_2_O_2_) accumulation under heat stress conditions. In addition, compared to its wild-type (WT) counterparts, *Arabidopsis thaliana* heterologously overexpresses *BcWRKY22*, improving thermotolerance. When the *BcWRKY22* transgenic root was obtained, under heat stress, the accumulation of H_2_O_2_ was reduced, while the expression of catalase 2 (*BcCAT2*) was upregulated, thereby enhancing CAT enzyme activity. Further analysis revealed that BcWRKY22 directly activates the expression of *BcCAT2* (*BraC08g016240.1*) by binding to the W-box element distributed within the promoter region of *BcCAT2*. Collectively, our findings suggest that BcWRKY22 may serve as a novel regulator of the heat stress response in non-heading Chinese cabbage, actively contributing to the establishment of thermal tolerance by upregulating catalase (CAT) activity and downregulating H_2_O_2_ accumulation via *BcCAT2* expression.

## 1. Introduction

Heat stress, caused by high temperatures, is a widely concerning agricultural problem. Short-term or sustained high temperatures can lead to physiological, morphological, and biochemical changes in plants, thus affecting a plant’s growth and development and causing sharp declines in economic yield. By using various genetic methods to develop crop plants with improved heat resistance, the adverse effects of heat stress can be mitigated [1]. Numerous vital cellular elements and metabolic processes involved in thermal responsive growth and the acquisition of heat resistance in plants have been identified through genetic, physiological, molecular, and biochemical research [2]. Plants develop defense mechanisms with which to cope with high temperatures, incorporating the buildup of heat shock proteins (HSPs) and the establishment of complex regulatory networks through transcription factors (TFs). Many mechanisms have developed in plants that exhibit extreme complexity and play essential roles in enhancing heat tolerance [1,3,4,5].

Elevated temperatures cause harm to plants through several mechanisms, such as inducing membrane damage, inactivating proteins, overproducing reactive oxygen species (ROS), and disrupting key metabolic functions [6,7]. ROS synthesis and clearance are balanced in healthy plants. However, under various stress conditions, this equilibrium is often disrupted, leading to the generation of excessive ROS and causing oxidative stress. This can be mitigated by ROS-detoxifying enzymes, such as catalase (CAT), superoxide dismutase (SOD), ascorbate peroxidase (APX), peroxide-reducing protein (PRX), and glutathione peroxidase (GPX). Alternatively, antioxidants like ascorbic acid and glutathione can also help clear excess ROS [8,9,10,11,12]. Although some transcription factors, such as HsfA1a, directly detect hydrogen peroxide and control the heat shock response gene expression in plants, our understanding of the relationship between hydrogen peroxide and various transcription factors that control thermotolerance is limited [13,14].

WRKY TFs can participate in a variety of adverse reactions during plant growth and development, and play an essential role in maintaining stress resistance in plants [15,16,17]. Previous research on WRKYs has focused on their role in coping with drought, cold, salt stress, and nutrient deficiencies [18,19,20,21,22,23]. Research on the heat resistance of WRKY transcription factors is relatively limited. After exposure to 45 °C for different periods of time, *wrky25* null mutants exhibited moderate heat sensitivity, lower germination rates, reduced hypocotyl and root growth, and increased electrical conductivity compared to their wild-type (WT) counterparts. In contrast, transgenic seeds overexpressing *AtWRKY25* showed stronger heat resistance [24]. *AtWRKY26*, *AtWRKY33*, and *AtWRKY25* positively regulate the co-operation between ethylene-activating proteins and heat-shock-protein-related signaling pathways in mediating the heat stress response in plants. These three proteins co-operate in a functional way to increase a plant’s ability to withstand heat [25]. The overexpression of *LlWRKY22* in lilies enhanced their heat resistance and upregulated the expression of the heat-responsive gene *LlDREB2B*. Conversely, the downregulation of *LlWRKY22* had the opposite effect [26]. LlWRKY39 can bind to the activation region of *LlMBF1c* and induce its expression, thereby enhancing the heat resistance of transgenic plants [27]. The positive regulation of the heat shock response (HSR) by AtWRKY39 involves the mediation of co-operative signaling pathways activated by salicylic acid and jasmonic acid [28]. A conserved WRKYGQK sequence and a zinc finger motif (CX_45_CX_22-23_HXH or CX_7_CX_23_HXC) are the distinguishing features of WRKY transcription factors, and WRKY proteins have the capacity to bind to the W-box (TGACC (A/T)), situated in the promoter region of their target genes, thereby regulating the downstream gene expression and modulating the stress response [15,29,30,31,32,33,34,35,36,37].

Non-heading Chinese cabbage (NHCC, *Brassica campestris* ssp. *chinensis*) is a significant vegetable that is grown extensively worldwide. High temperature is the main limiting factor for vegetable production, and non-heading Chinese cabbage does not grow well at high temperatures (over 25 °C) [38]. Exposure to high temperatures directly leads to a decrease in yield and affects the quality of the food, while heat stress also affects photosynthesis and can even affect downy mildew, soft rot, viral diseases, and other diseases [39].

In this study, a heat-induced member of the WRKY-IIe family, *BcWRKY22*(*BraC09g001080.1*), was isolated and identified from NHCC. According to subcellular localization studies, BcWRKY22 is situated in the nucleus, and *BcWRKY22* exhibits activation in yeast. In addition, the transient overexpression of *BcWRKY22* improved the heat tolerance of NHCC leaves, and the overexpression in the roots enhanced root heat tolerance. Moreover, *Arabidopsis thaliana* also exhibited increased heat tolerance when *BcWRKY22* was overexpressed. Further analysis showed that BcWRKY22 directly activates the expression of *BcCAT2*(*BraC08g016240.1*), which improves CAT activity and reduces H_2_O_2_ content, thereby positively regulating thermotolerance.

## 2. Materials and Methods

### 2.1. Plant Materials and Growth Conditions

The cultivated variety of non-heading Chinese cabbage, ‘NHCC001’, was utilized in this experiment under a 16 h light/8 h darkness cycle at a temperature of 24 °C/18 °C with a humidity level of 55%. *Arabidopsis thaliana* (Col) and *N. benthamiana* were raised in potting medium within a growth room set to maintain a temperature of 24 °C, with a photoperiod lasting for 16 h.

### 2.2. Clone BcWRKY22 from NHCC001

Total RNA was isolated from the leaves of NHCC001 plants four weeks after germination. The NHCC’s total RNA was isolated using the RNAsimple Total RNA Kit (TIANGEN, Beijing, China) and reverse-transcribed with Hifair^®^ III 1st strand cDNA synthesis superMIX for qPCR (11141ES60, Yeasen, Shanghai, China). A designed primer was used to amplify the open reading frame (ORF) of *BcWRKY22*. Primers for gene cloning were shown in Supplementary Table S1.

### 2.3. Subcellular Localization of BcWRKY22

To create the 35S:*BcWRKY22*-GFP construct, the coding region of BcWRKY22 was inserted into the pRI101-GFP vector. The reconstructed plasmid and empty vector were introduced into *Agrobacterium* GV3101, and individual colonies were grown in Luria broth with 50 µg·mL^−1^ rifampicin and 50 µg·mL^−1^ kanamycin supplemented at 28 °C for 20 h. Following PCR identification, the bacterial strains were cultured in 5 mL of Luria broth supplemented with 50 µg·mL^−1^ rifampicin and 50 µg·mL^−1^ kanamycin at 28 °C for 16–20 h. The bacterial solutions were then centrifuged, resuspended in infiltration medium (10 mM MgCl_2_, 10 mM MES, and 150 µM Acetosyringone; pH adjusted to 5.7), and incubated at room temperature for a minimum of four hours until they reached OD600 values between 0.8 and 1.0. Finally, the Agrobacterium solution was injected into the backside of the tobacco leaves using pressure osmosis. After a 48 h infiltration period, the leaves were excised and observed for GFP and RFP fluorescence using a laser scanning confocal microscope (LSM800, Zeiss, Oberkochen, Germany). Three independent replicate experiments were performed to obtain a representative image.

### 2.4. Transactivation Activity Assay of BcWRKY22 in Yeast

The open reading frame of *BcWRKY22* was cloned into the pGBKT7 vector. The recombinant constructs, pGBKT7-*BcWRKY22* and pGBKT7-GAL4, were altered into an AH109 yeast strain. Transformants were selected on SD medium lacking tryptophan at 28 °C for 3 days. Positive clones were screened on SD medium, without tryptophan and histidine, containing X-α-gal at 28 °C for 3 days. Three independent replicates were conducted to make the results representative. Supplementary Table S1 showed the primers used for vector construction.

### 2.5. Promoter Activity Analysis of BcWRKY22

The 2000 bp fragment of the *BcWRKY22* promoter was isolated and ligated to a pGreenII-0800-LUC vector using recombinant ligase (Vazyme, Nanjing, China). The final construct and its control were transformed into *A. tumefaciens* strain GV3101 (pSoup) for infiltration into tobacco leaves. After 48 h, half of the infiltrated leaves underwent heat stress at 40 °C for 1.5 h, followed by recovery at 24 °C for 12 h. Subsequently, the fluorescence signal was detected using a plant living imaging system (Berthold, Stuttgart, Germany). The activities of firefly luciferase (LUC) and Renilla luciferase (REN) were measured using a luciferase reporter assay system (Yeasen, Shanghai, China). Three replicate experiments were conducted to obtain representative results. Supplementary Table S1 shows the primers used for vector construction.

### 2.6. Heat Stress Treatment of NHCC001

To assess gene expression levels in response to heat shock (HS), 4-week-old ‘NHCC001’ plants were subjected to a temperature of 40 °C for varying durations of 0, 1, 3, 6, 12, and 24 h, a set of experiments treated at room temperature for the same time under dark conditions as a parallel control. The heat treatments were administered in a light-free incubator (DRX-160B-LED, Ningbo, Nanjing, China). After the treatment, leaf samples were collected for qRT-PCR analysis. We subtracted the corresponding room temperature data from all heat treatment data, and then performed calculations according to the formula 2^−ΔΔCT^ with 0 h as the basis. Three independent replicate experiments were used for statistical analysis via Student’s *t*-test. Supplementary Table S1 shows the primers used for vector construction.

### 2.7. Transient Overexpression Assay in NHCC001 Leaves

Bacterial cultures expressing pRI101-GFP or pRI101*-BcWRKY22-*GFP were resuspended using an infiltration buffer (10 mM MgCl_2_, 10 mM MES, and 150 µM Acetosyringone; pH adjusted to 5.7) and incubated at 24 °C for 4 h under dark conditions. NHCC001 was employed for transient overexpression. Subsequently, leaf discs, each with a diameter of 1 cm, were excised using a hole punch and infiltrated with bacterial solutions under negative pressure (−0.8 MPa, 30 min). The infiltrated discs were rinsed with sterile water and placed on 0.4% agar medium at 24 °C for 72 h. For heat shock treatment, the discs were exposed to 40 °C for 16 h. Heat stress can impact chlorophyll content, resulting in a reduction thereof. The extent of damage to chlorophyll is indicative of heat tolerance [40,41,42,43,44,45,46,47,48,49,50]. The activities of SOD and CAT enzymes were assessed following a 3 h heat treatment, while the levels of Malondialdehyde (MDA) and H_2_O_2_ were determined after a 16 h period. The SOD activities of treated and untreated leaves were determined with the NBT method using a Superoxide Dismutase (SOD) Activity Assay Kit (Solarbio, Beijing, China). Using the Titanium sulfate method, the H_2_O_2_ concentration was measured with a Hydrogen Peroxide (H_2_O_2_) Content Assay Kit (Solarbio, Beijing, China). A Malondialdehyde (MDA) Content Assay Kit (Solarbio, Beijing, China) was used with the TBA method to measure the MDA content of treated and untreated leaves, and the CAT activities of treated and untreated leaves were determined using the ultraviolet absorption method with a Catalase (CAT) Activity Assay Kit (Solarbio, Beijing, China). Three independent technical replicate experiments were conducted for each of the three biological replicates.

### 2.8. Agrobacterium-Mediated Transformation of Arabidopsis

The lack of a stable genetic transformation system for NHCC has hindered the acquisition of NHCC transgenic plants. Therefore, transgenic *Arabidopsis* plants were generated using the *Agrobacterium*-mediated floral dipping method [51]. The CDS of *BcWRKY22* was cloned into the pRI101 vector and subsequently transformed into *Agrobacterium* GV3101 for recombinant plasmid construction.

The T0 seeds were examined on MS medium with timentin and hygromycin, and positive T1 lines were identified using PCR. Subsequent generations (T2/T3) were identified using the same methods, and the stable T3 generation was used to study *BcWRKY22* function. Three independent replicate experiments were performed to assess reliable results. Supplementary Table S1 shows the primers used for vector construction.

### 2.9. Agrobacterium-rhizogenes-Mediated Transformation of Roots

Transgenic roots were obtained according to the methods reported by Chen [52]. The ‘NHCC001’ seeds were treated with 75% ethanol for 5 min and 10% sodium hypochlorite for 12 min, washed three times with ddH_2_O, and then placed on germination medium (1/2 MS, pH 5.8) and kept in a cultural space at 24 ± 2 °C under a light cycle of 16 h light and 8 h dark for 5–7 days (light intensity: 3000 lx). *BcWRKY22* overexpression vector 2300-*BcWRKY22* was introduced into the *Agrobacterium rhizogenes* MSU440 strain, which was developed overnight. The bacterial solution was then centrifuged at 6000 rpm for 10 min and the supernatant was abandoned. A co-culture liquid medium (MS-diluted tenfold (pH5.2) + acetosyringone (100 μM)) was added to make OD600 = 1.0 and placed at 28 °C and 200 rpm for three hours to form the infection medium. We removed the main roots of the seedling and soaked it in infection medium for 10 min. After washing it three times with ddH_2_O, we placed it in MS medium containing timentin (250 mg/L). The hairy roots grew out after 25–35 days and were used for subsequent experiments following identification. The H_2_O_2_ content, MDA content, and CAT activity of treated and untreated leaves were determined using a kit (Solarbio, Beijing, China). Following a 3 h heat treatment, the activity of CAT enzymes was assessed. Additionally, the levels of MDA and H_2_O_2_ were examined after a 16 h duration. With the titanium sulfate method, a Hydrogen peroxide (H_2_O_2_) Content Assay Kit (Solarbio, Beijing, China) was used to measure the H_2_O_2_ concentration; a Malondialdehyde (MDA) Content Assay Kit (Solarbio, Beijing, China) was used to measure the MDA content of treated and untreated leaves using the TBA method. Using ultraviolet absorption method, the CAT activity of treated and untreated leaves was determined using a microplate reader with a Catalase (CAT) Activity Assay Kit (Solarbio, Beijing, China). Supplementary Table S1 shows the primers used for vector construction. Each of the three biological replicates was subjected to three independent technical replicates.

### 2.10. Quantitative Real-Time PCR Analysis

The RNAsimple Total RNA Kit (TIANGEN, Beijing, China) was chosen for extracting total RNA from NHCC and *Arabidopsis thaliana*, which were subsequently reverse-transcribed using a Hifair^®^ Ⅲ 1st strand cDNA synthesis superMIX for qPCR (11141ES60, Yeasen, Shanghai, China). The Hieff^®^ qPCR SYBR Green Master Mix (11202ES03, Yeasen, Shanghai, China) was used for real-time quantification PCR on QuantStudio 5 (ABI, Los Angeles, CA, USA), with *BcGAPC* and *AtActin* as internal standards. Data analysis was performed using the 2^−ΔΔCT^ method with three repeats. For each of the three biological replicates, three separate technical replicates were employed. Primers used for qRT-PCR are listed in Supplementary Table S1.

### 2.11. Y1H Assay

The *BcCAT2* promoters were cloned into the pLacZi vector, while the *BcWRKY22* ORF was fused into the pJG vector to create pJG*-BcWRKY22*. The empty pJG vector served as a negative control. Reconstructed vectors were co-transformed with Yeast EGY48 strain cells for a Y1H assay. Transformants were selected on Ura-/Trp-deficient SD medium at 28 °C, and binding activity was assessed through x-gal color change. To evaluate the reliability of the results, three independent replicate experiments were conducted. Supplementary Table S1 shows the primers used for vector construction, and the analysis of promoter elements was shown in Supplementary Table S2.

### 2.12. Electrophoretic Mobility Shift Assay (EMSA)

The open reading frame of *BcWRKY22* was inserted into the pMAL-MBP vector and introduced into *Escherichia coli* Rosetta (DE3). Upon induction with isopropyl-β-D-thiogalactoside (IPTG), the MBP-BcWRKY22 fusion protein was generated. The EMSA probe primers were biotin-labeled.

Then, EMSA was conducted using the LightShift Chemiluminescent EMSA Kit (Beyotime, Shanghai, China). The LightShift Chemiluminescent EMSA Kit (Beyotime, Shanghai, China) was utilized to perform EMSA. Three independent replicate tests were conducted to evaluate a representative image. Supplementary Table S1 displays the primers employed for generating the EMSA probe.

### 2.13. Dual-Luciferase Reporter Assay

The *BcWRKY22* ORF was subsequently inserted into the pRI101 vector, giving rise to the pRI101-*BcWRKY22* effector vector, while the empty vector stood as a negative control. The *BcCAT2* promoters were fused with the pGreenII0800-LUC vector to create LUC reporters, which were then introduced into *A. tumefaciens* GV3101 (pSoup) cells and injected into tobacco leaves with mixtures of bacteria that express various arrangements of reporters and effectors. The LUC signal was observed in the infiltrated leaves and measured 48 h later. The enzymatic activities of LUC and REN were determined using the Dual-Luciferase Reporter Gene Assay Kit (Yeasen, Shanghai, China). There were three distinct technical replicates used for each of the three biological replicates.

## 3. Results

### 3.1. A WRKY-IIe Member BcWRKY22 Transcript Increased in Heat-Tolerant Cultivar NHCC001 after Heat Treatment

**Figure 1:**
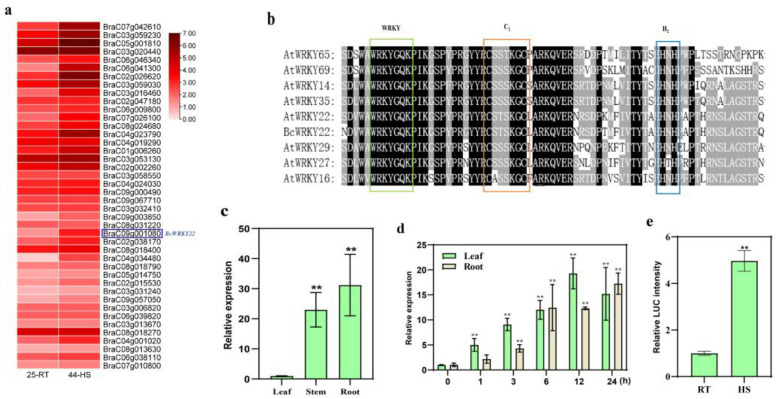
The gene *BcWRKY22* belongs to the WRKY-IIe subgroup and exhibits heat-inducible expression. (**a**) WRKY expression heat map in NHCC leaves under heat stress (HS). The heat map visualizes the expression pattern of WRKYs on the basis of the heat-treated transcriptome of NHCC leaves; the blue box indicates *BcWRKY22*; 25-RT: normal condition (25 °C); 44-HS: exposed to 44 °C for 4 h. (**b**) Multiple alignments of the conserved domains in the WRKY-IIe group. Analogous amino acids are tinted in gray, while identical amino acids are displayed with a black backdrop. The WRKY motif is exhibited with a green border, while the zinc-finger structures are depicted with orange and blue borders. (**c**) The expression of *BcWRKY22* in NHCC leaves, stems, and roots. The values are expressed as the means ± SD of three replicates (Student’s *t*-test, ** *p* < 0.01). (**d**) The expression of *BcWRKY22* in NHCC leaves and roots under heat stress (RT: 24 °C; HS: 40 °C) exposure of differing durations. Bars indicate means ± SD from three replicates (Student’s *t*-test, ** *p* < 0.01; all treatments compared with 0 h). (**e**) Quantitation of LUC intensity in tobacco leaves. The *BcWRKY22* promoter activity was assessed using the LUC reporter assay in tobacco leaves under room temperature (24 °C) and heat stress (HS: 40 °C, 3 h; followed by recovery at 24 °C for 12 h). Bars indicate means ± SD from three replicates (Student’s *t*-test, ** *p* < 0.01).

The investigation of WRKY gene expression in the transcriptome (the transcriptome data have been determined by previous researchers and the data have been published (http://nhccdata.njau.edu.cn/ (accessed on 2 July 2021)) [39] of non-heading Chinese cabbage NHCC001 revealed that *BcWRKY22* exhibited a differential expression in response to high-temperature conditions (Figure 1a). The cDNA sequence of *BcWRKY22* contains an 897 bp Open Reading Frame (ORF) encoding 298 amino acid proteins. The phylogenetic tree containing all WRKY proteins in *Arabidopsis thaliana* suggests that *BcWRKY22* belongs to the WRKY-IIe family and is closely related to *AtWRKY22*, *AtWRKY29*, and *AtWRKY27* (Figure S1). Their sequences have similar features, including a WRKYGQK sequence and a C_2_H_2_ (C-X_5_-C-X_23_-H-X_1_-H) zinc-binding motif (Figure 1b).

In order to investigate the expression level of *BcWRKY22* in various tissues, RT-qPCR was performed on the roots, stems, and leaves of NHCC001; the results showed that the expression levels of *BcWRKY22* in the roots and stems were significantly higher than that in the leaves, and the expression level was the highest in the roots (Figure 1c). Meanwhile, to detect the *BcWRKY22* expression under high-temperature conditions, NHCC001 plants were exposed to 40 °C for heat stress (HS) treatment (44 °C can cause irreversible damage to plants; below 40 °C, the heat damage phenotype is not obvious, and there is no significant difference in gene and indicator changes compared to 40 °C, so we used 40 °C instead of 44 °C for the transcriptome screening genes). Based on the RT-qPCR results, and compared with the treatment at 24 °C, *BcWRKY22* expression was rapidly induced in the leaves after 1 h of HS and in the roots after 3 h, and both stayed at high levels even 24 h after HS (Figure 1d). Subsequently, the promoter activity of *BcWRKY22* was analyzed using the LUC reporter assay. The results showed that the *BcWRKY22* promoter-driven LUC reporter signal in tobacco leaves was significantly increased after HS treatment (Figure 1e). Thus, *BcWRKY22* is a heat-responsive WRKY-IIe gene in NHCC001.

### 3.2. Localization in the Subcellular Region and Transactivation Activity of BcWRKY22

**Figure 2:**
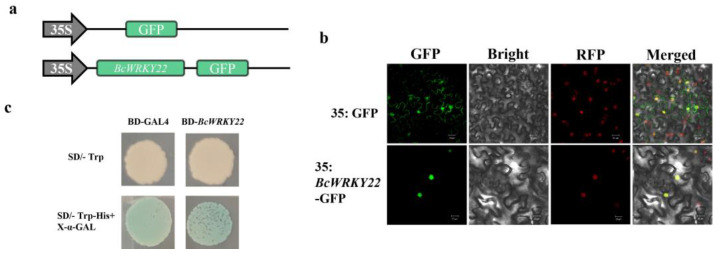
Transactivation activation assay and subcellular localization of BcWRKY22. (**a**) The constructs for the subcellular localization assay. (**b**) Confocal microscopy was used to examine BcWRKY22 localization, which was found in the nucleus. *BcWRKY22*-GFP and the nuclear marker mCherry overlapped in the nucleus. Bars = 20 μm. (**c**) Transcriptional activation activity of *BcWRKY22* in yeast. The transformants were selected on SD-W medium (devoid of Trp), while the culture of transformants was assessed on SD-WH medium (lacking Trp/His) supplemented with X-α-GAL.

Through the analysis of a BcWRKY22-GFP fusion protein, the subcellular localization of BcWRKY22 was examined. The full-length ORF of *BcWRKY22* was amplified to the N-terminal of the GFP reporter protein of the pRI101 vector, driven by the CaMV 35S promoter, generating a recombinant construct *BcWRKY22*-GFP. We used an RFP nuclear signaling peptide as a positive control. Laser confocal microscopy showed that *BcWRKY22* fusion protein fluorescence overlaps with RFP fluorescence, suggesting that *BcWRKY22* is located in the nucleus (Figure 2a,b). In addition, *BcWRKY22* showed transactivation activity in yeast cells (Figure 2c).

### 3.3. BcWRKY22 Overexpression Increases the Thermotolerance of Non-Heading Chinese Cabbage

**Figure 3:**
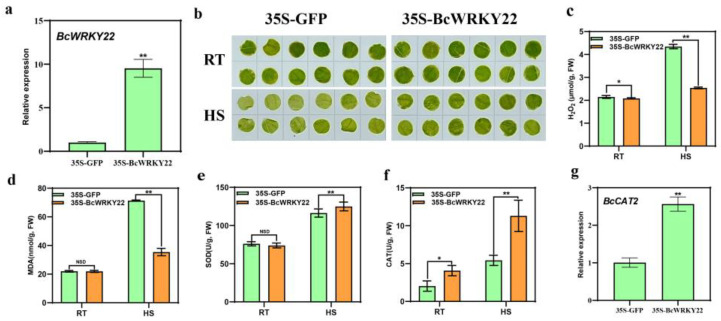
*BcWRKY22* transient expression positively regulates NHCC thermotolerance. (**a**) Relative expression levels of *BcWRKY22* in transient transferred lines. Values are presented as the means ± SD of three replicates (Student’s *t*-test, ** *p* < 0.01); the 35S-BcWRKY22 compared with the control (35S-GFP). (**b**) The leaf disc phenotypes of NHCC were observed at room temperature (RT: 24 °C), and after exposure to heat stress (HS: 40 °C, 16 h). (**c**) H_2_O_2_ content determined at RT and under heat stress (HS: 40 °C, 16 h)). Values are presented as the means ± SD of three replicates (Student’s *t*-test, * *p* < 0.05; ** *p* < 0.01; the 35S-BcWRKY22 compared with the control (35S-GFP) at RT or under the HS condition). (**d**) The MDA content of discs at RT and under HS. The data are presented as the means ± SD of three repeated experiments (Student’s *t*-test, ** *p* < 0.01; NSD, no significant difference; the 35S-BcWRKY22 compared with the control (35S-GFP) at RT or under the HS condition). (**e**) The SOD content of discs at 24 °C (RT) and under HS (40 °C, 3 h). The data are represented as the means ± SD of three replicates (Student’s *t*-test, ** *p* < 0.01; NSD, no significant difference; the 35S-BcWRKY22 compared with the control (35S-GFP) at RT or under the HS condition). (**f**) The CAT content of discs at 24 °C (RT) and under HS (40 °C, 3 h). The data are presented as the means ± SD of three replicated experiments (Student’s *t*-test, * *p* < 0.05; ** *p* < 0.01; the 35S-BcWRKY22 compared with the control (35S-GFP) at RT or under the HS condition). (**g**) Relative expression levels of *BcCAT2* in transient transferred lines. All data are the averages of three independent experiments; each error bar indicates mean ± SD from three replicates; ** *p* < 0.01 (Student’s *t*-test).

To investigate the function of *BcWRKY22* in vivo, we overexpressed *BcWRKY22* in NHCC001 leaves with a transient transformation assay. The RT-qPCR results showed that the expression level of *BcWRKY22* in leaves was significantly higher than that in the control (Figure 3a). Then, after HS treatment, we found that the green degree of *BcWRKY22* transiently transformed round hole leaves was higher than that in the control (Figure 3b). Interestingly, the H_2_O_2_ content of the overexpressed *BcWRKY22* was lower at 24 °C than that of the control; meanwhile, after HS, the increased degree of H_2_O_2_ content in the *BcWRKY22*-overexpressed leaves was significantly lower than that of the control (Figure 3c). Additionally, the overexpression of *BcWRKY22* in leaves had no impact on the MDA content or SOD activity at 24 °C. After HS, the value for MDA was significantly higher in the control leaves than in the *BcWRKY22*-overexpressed leaves, and the SOD activity was significantly lower in the control leaves than in the *BcWRKY22*-overexpressed leaves (Figure 3d,e). Furthermore, the CAT activity of the overexpressed *BcWRKY22* was higher at 24 °C than in the control. After HS, the CAT activity of the *BcWRKY22*-overexpressed leaves increased more (Figure 3f). Therefore, we determined the expression level of *BcCAT2*, a gene related to catalase (CAT) activity, and the expression level of *BcCAT2* in transient *BcWRKY22*-overexpressed leaves was noticeably higher than those of the control (Figure 3g). These results suggested that *BcWRKY22* overexpression protects NHCC001 leaves from high-temperature stress and improves their heat resistance; moreover, *BcWRKY22* may improve heat resistance by increasing CAT activity to reduce H_2_O_2_ content in NHCC001.

### 3.4. Overexpression of BcWRKY22 Increased the Thermotolerance of Transgenic Arabidopsis thaliana

**Figure 4:**
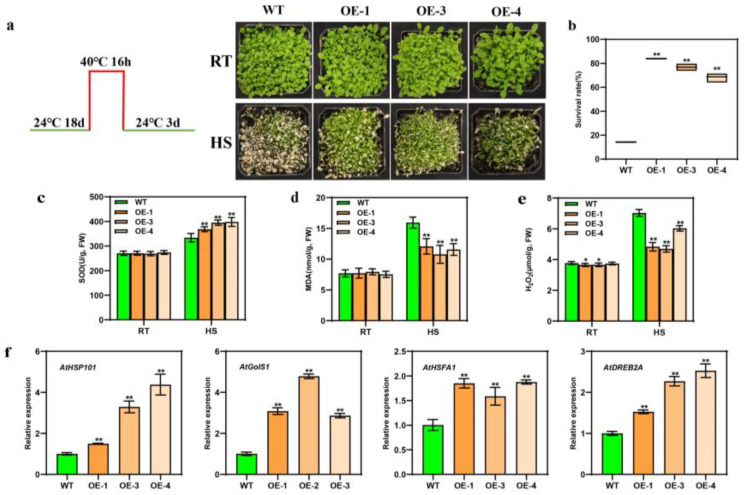
Overexpressing *BcWRKY22* enhances thermotolerance in *Arabidopsis thaliana* and upregulates the expression of heat-responsive genes. (**a**) Culture temperature and time setting for *Arabidopsis thaliana*, and the comparison between overexpressed *Arabidopsis* and WT phenotypes at RT (24 °C) and HS (40 °C, 16 h; restored for 3 days at 24 °C). (**b**) The survival rates of overexpressed *Arabidopsis* specimens and the WT control after HS (40 °C, 16 h) and restoration over 3 daysat 24 °C. Values are presented as the means ± SD of three replicates (Student’s *t*-test, ** *p* < 0.01). (**c**) The SOD content values of the WT control and the three transgenic lines at RT (24 °C) and under HS (40 °C, 3 h). The data are indicated as the means ± SD of three replicates (Student’s *t*-test, ** *p* < 0.01). (**d**) The MDA content values of the WT control and the three transgenic lines at RT (24 °C) and under HS (40 °C, 16 h). The data are displayed as the means ± SD of three replicates. (Student’s *t*-test, ** *p* < 0.01). (**e**) H_2_O_2_ content values of the WT control and the three transgenic lines, determined at 24 °C (room temperature (RT)) and under heat stress (HS, 40 °C, 16 h)). The data are presented as the means ± SD of three replicates (Student’s *t*-test, * *p* < 0.05; ** *p* < 0.01). (**f**) Relative expressions of *AtHSP101*, *AtGolS1*, *AtHSFA1*, and *AtDREB2A* in the three transgenic lines and the WT control. All data are represented as the means ± SD of three replicates (Student’s *t*-test, ** *p* < 0.01).

We allogenically transformed the *BcWRKY22* gene into *Arabidopsis thaliana*, and three homozygous T3 transgenic *Arabidopsis* lines were identified (Figure S2). Then, three transgenic lines (OE-1, OE-3, and OE-4) and a WT control were treated at 40 °C for 16 h and recovered for 3 days (Figure 4a). Under heat stress (HS) conditions, the WT control’s survival rate was noticeably lower than that of the three transgenic lines (Figure 4b). Meanwhile, the overexpression of *BcWRKY22* in *Arabidopsis thaliana* did not affect the SOD activity or MDA content at 24 °C (RT). After HS, the SOD activity was significantly lower in the WT control than in those of the three transgenic lines, and the value for MDA was significantly higher in the WT control than in those of the three transgenic lines (Figure 4c,d). This outcome was consistent with the result of transient overexpression *BcWRKY22*, in that the H_2_O_2_ content values were lower at 24 °C in the three transgenic lines than in the WT control. After HS, the increased degree of H_2_O_2_ content values in the three transgenic lines was significantly lower than that of the WT (Figure 4e). Furthermore, the expression levels of HS-induced genes *AtHSP101*, *AtGolS1*, *AtHSFA1*, and *AtDREB2A* were significantly increased in transgenic *Arabidopsis* (Figure 4f). These results suggested that *BcWRKY22* overexpression improved the heat tolerance of transgenic *Arabidopsis*.

### 3.5. BcWRKY22 Expression in NHCC Roots Increased the Thermotolerance of Roots

**Figure 5:**
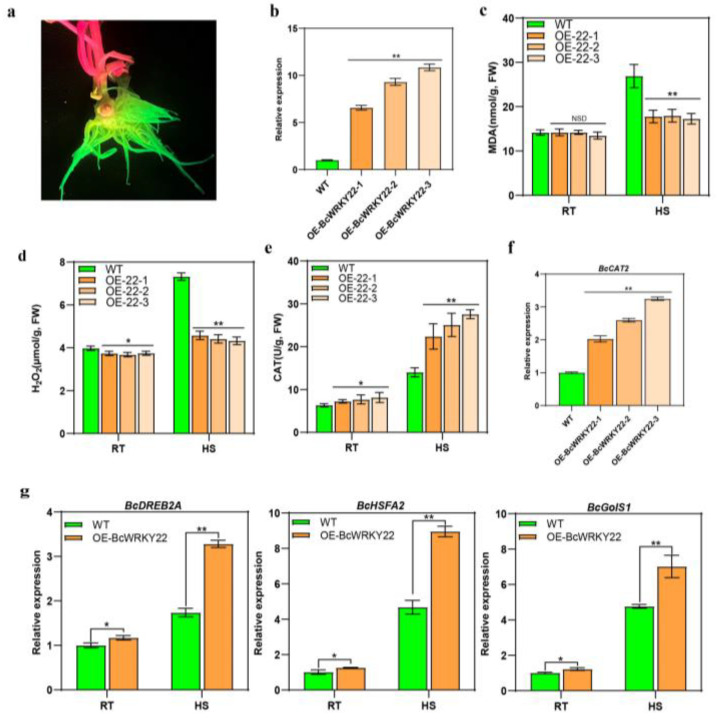
Overexpression of *BcWRKY22* in NHCC roots enhance heat resistance and induces the upregulation of genes associated with heat response. (**a**) LUYOR-3415-observed GFP fluorescence photograph of the NHCC roots. (**b**) The expression of *BcWRKY22* in the three overexpressed root systems and in the WT control. The error bars indicate the means ± SD from three repetitions (Student’s *t*-test, ** *p* < 0.01). (**c**) The MDA content values in the WT control and the three overexpressed root systems at RT (24 °C) and under HS (40 °C, 16 h). The values are presented as the means ± SD of three repetitions (Student’s *t*-test, ** *p* < 0.01; NSD, no significant difference). (**d**) H_2_O_2_ content values of the WT control and the three overexpressed root systems were determined at 24 °C (room temperature (RT) and under heat stress (HS, 40 °C, 16 h)). Values are represented as the means ± SD of three repeated experiments (Student’s *t*-test, * *p* < 0.05; ** *p* < 0.01). (**e**) The CAT content values of the three overexpressed root systems and the WT control at RT (24 °C) and under HS (40 °C, 3 h). The data are represented as the means ± SD of three repeated experiments (Student’s *t*-test, * *p* < 0.05; ** *p* < 0.01). (**f**) Relative expression level of *BcCAT2* in the three overexpressed root systems and the WT control. All data are the averages of three independent experiments, and error bars indicate the means ± SD from three replicates, ** *p* < 0.01 (Student’s *t*-test). (**g**) *BcDREB2A*, *BcHSFA2*, and *BcGolS1* relative expression levels in the three overexpressed root systems and the WT control. (RT, 24 °C; HS, 40 °C). All data are the averages of three independent experiments, and error bars indicate the means ± SD from three replicates (Student’s *t*-test, * *p* < 0.05; ** *p* < 0.01).

Since the expression of *BcWRKY22* was at its highest in roots (Figure 1c), we obtained transgenic roots using *Agrobacterium rhizogenes* in NHCC, and the GFP fluorescence of roots was observed with LUYOR 3415, a fluorescent protein excitation source, to ensure that *BcWRKY22*-overexpressed roots were obtained (Figure 5a). An RT-qPCR analysis showed that *BcWRKY22* was overexpressed in the three root systems (OE-*BcWRKY22*-1, OE-*BcWRKY22*-2, and OE-*BcWRKY22*-3), and their gene expression levels were higher than that of the WT control (Figure 5b). Then, we treated the WT control and the three overexpressed roots with heat stress (HS). The overexpression of *BcWRKY22* in the three root systems did not affect their MDA content values at 24 °C, while the values of MDA were significantly higher in the WT control than in the *BcWRKY22*-overexpressed roots under HS (Figure 5c). Meanwhile, we found that the H_2_O_2_ content values of the three transgenic roots were lower than that of the WT control at 24 °C, and the increased degree of H_2_O_2_ content values in the three transgenic roots was markedly lower than that of the WT control after HS (Figure 5d). This conclusion is compatible with the results of the transient expression and overexpression in *Arabidopsis thaliana*, indicating that the overexpression of *BcWRKY22* can affect the content of H_2_O_2_. Furthermore, the CAT activity of the *BcWRKY22*-overexpressed root systems was higher at 24 °C than that of the WT control. After HS, compared with the WT control, the increase in CAT activity in the *BcWRKY22*-overexpressed roots was significantly upregulated (Figure 5e). In addition, the expression of *BcCAT2* was significantly increased in the *BcWRKY22*-overexpressed roots (Figure 5f). In order to further confirm that the overexpression of *BcWRKY22* can improve the thermotolerance of roots, we determined the expression levels of the heat response genes *BcDREB2A*, *BcHSFA2*, and *BcGolS1*. Their expression levels increased significantly in transgenic roots. Moreover, after HS, the expression of the three genes increased even more (Figure 5g). In summary, these results suggest that *BcWRKY22* overexpression improves the thermotolerance of transgenic roots. At the same time, *BcWRKY22* may regulate the expression level of *BcCAT2* to improve CAT activity and reduce the accumulation of H_2_O_2_.

### 3.6. BcWRKY22 Binds to the Promoter of BcCAT2 and Activates Its Expression

**Figure 6:**
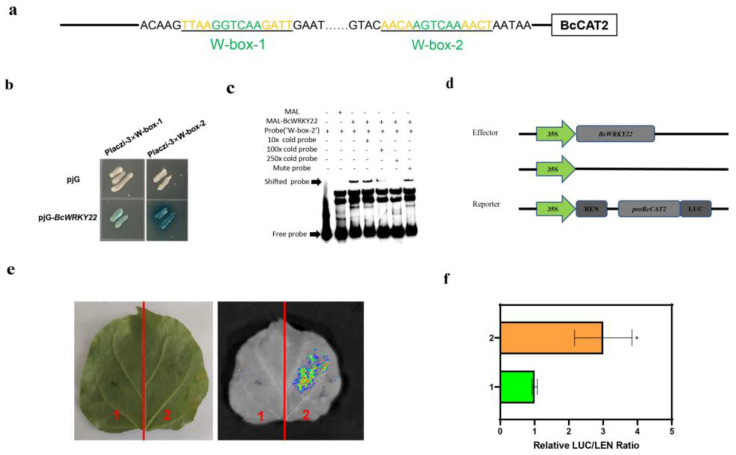
BcWRKY22 activated *BcCAT2* expression directly. (**a**) The *BcCAT2* promoter is shown in this diagram, in which the W-box elements are highlighted in green. Black underlining indicates truncated fragments for the yeast one-hybrid (Y1H) assay. (**b**) A Y1H assay for BcWRKY22 and the fragments of the *BcCAT2* promoter. A color change on Ura-/Trp-deficient SD medium was used to measure fragment activity following the addition of x-gal. Based on three replications, this is a representative image. (**c**) EMSA was employed to identify the direct interaction between BcWRKY22 and the W-box-2 region within the *BcCAT2* promoter. The EMSA of the biotin-labeled oligonucleotide was derived from the putative BcWRKY22 binding site of the *BcCAT2* promoter in the presence or absence of a cold competitor and mutated probe. Crude BcWRKY22 protein (3 μg) was incubated with 100 nM biotin-labeled probes. To assess the competition, cold competitor and mutated probes were added at different concentrations (10×, 100×, and 250×) during the experiment. Presence (+) or absence (−) of the components is indicated at the top. (**d**) A dual-luciferase reporter assay’s supporting constructs. (**e**) Finding the LUC signal in tobacco leaves. Based on three experiments, this is a representative image. 1: *pro-BcCAT2*+pRI101 empty; 2: *pro-BcCAT2*+pRI101-*BcWRKY22*. (**f**) Measurement of LUC intensity in the dual-luciferase reporter assay. Values are represented as the means ± SD of three replications (Student’s *t*-test, * *p* < 0.05).

Based on our findings that *BcCAT2* being activated in both *BcWRKY22*-overexpressed NHCC leaves and *BcWRKY22*-overexpressed transgenic roots (Figure 3g and Figure 5f), we speculated that BcWRKY22 might increase CAT activity and reduce H_2_O_2_ accumulation by regulating the expression level of *BcCAT2*. Meanwhile, we found that *BcCAT2* had two common W-boxes in its promoter (Figure 6a). A yeast one-hybrid (Y1H) assay showed that BcWRKY22 could strongly activate 3 × W-box-2; however, the activation ability of 3 × W-box-1 is weak (Figure 6b). Therefore, we chose W-box-2 for the “Electrophoretic mobility shift assay” (EMSA). The EMSA results also revealed that MBP-BcWRKY22 could bind to the W-box-2 tandem element of the *BcCAT2* promoter, while adding W-box-2, mutated from AGTCAA to AAAAAA, would not affect the binding, thus suggesting that BcWRKY22 may play a direct regulatory role in the expression of *BcCAT2* (Figure 6c). In addition, we constructed vectors for a dual-luciferase reporter assay (Figure 6d); in this experiment, we used the full-length promoter of *BcCAT2*. Effector reporter tests showed a lower LUC signal in the leaves of *N. benthamiana* co-transformed with the control combination, but a significantly increased LUC signal and a significantly higher LUC/REN ratio after co-transformation with *BcWRKY22* and pro*BcCAT2*-LUC (Figure 6e,f). Taken together, these results suggest that BcWRKY22 can directly activate *BcCAT2* by binding to its promoter.

## 4. Discussion

Previous research found that the *AtWRKY22* transcription factor may be involved in multiple regulatory pathways, which play important roles in regulating the growth and development of *Arabidopsis thaliana*. Dark-treated *AtWRKY22* overexpression and knockout lines caused age-related genes, and increased and decreased expression levels, which led to accelerated and delayed aging phenotypes, respectively [53]. Meanwhile, in coping with biostress, *AtWRKY22* not only acts downstream of the flagellin receptor FLS2 and participates in activating the MAPK cascade and confers resistance against bacterial and fungal pathogens [54], but also enhances the susceptibility to aphids and modulates the signaling pathways of salicylic acid and jasmonic acid [55]. Additionally, among other species, *NbWRKY22* and *NbWRKY25* fully activate immunity against bacterial patterns, effectors, and non-bacterial defense triggers [56], while overexpressed *LlWRKY22* in lily induced the expression of heat-related gene *LlDREB2B*, thereby enhancing heat tolerance [26]. However, the role of *BcWRKY22* in NHCC remains unexplored. We identified *BcWRKY2*2, a member of the WRKY-IIe subgroup with a classical WRKY domain. Its expression is induced by high temperature, and it exerts a function in the nucleus in response to HS. Transcriptomic analysis revealed that *BcWRKY22* is a heat-responsive gene in NHCC. Furthermore, we observed a significant upregulation of both *AtDREB2A* and *BcDREB2A* subsequent to the overexpression of *BcWRKY22* (Figure 4f and Figure 5g). *BcWRKY22* has the potential to enhance heat resistance by regulating *BcDREB2A* in NHCC. Meanwhile, we observed that the overexpression of *BcWRKY22* in NHCC cells resulted in an increased activity of the CAT enzyme (Figure 3f and Figure 5e), indicating its potential to mitigate H_2_O_2_ toxicity and enhance heat resistance by modulating CAT activity.

Our findings that heat shock induces the expression of *BcWRKY22* suggests that *BcWRKY22* may be involved in regulating the heat shock response (HSR) (Figure 1). Therefore, we elucidated the role of *BcWRKY22* in HSR. The overexpression of *BcWRKY22* enhanced the thermotolerance of NHCC leaves and roots, as well as those of *Arabidopsis* (Figure 3, Figure 4 and Figure 5). Several heat shock response genes, including *AtHSFA1*, *AtDREB2A*, *AtHSP101*, and *AtGolS1*, exhibited significant upregulation in the transgenic line (Figure 4). Similarly, *BcHSFA1*, *BcDREB2A*, and *BcGolS1* were also significantly upregulated in transgenic roots (Figure 5). Given their active roles in enhancing heat tolerance mechanisms within plants, it is plausible that the increased expression of these genes may contribute to an overall improvement in thermal stress resistance [57,58,59,60,61,62,63,64,65]. At the same time, the MDA content, H_2_O_2_ content, and CAT enzyme activity are also important indicators of heat resistance in plants [57,66,67,68,69,70,71,72,73,74,75]. The overexpression of *BcWRKY22* in plants demonstrated an enhancement in heat resistance through the observed physiological changes.

In plants, reactive oxygen species (ROS) are considered a pivotal signaling component for the crosstalk between biological and abiotic stress responses. Among the ROS, hydrogen peroxide (H_2_O_2_) stands out as the most stable one, acting as a crucial signaling molecule in defense responses against pathogens and abiotic stresses such as heat [76,77,78,79]. Previous research claimed that reduced activity of the antioxidant enzymes, particularly catalase (CAT), superoxide dismutase (SOD), and glutathione peroxidases (GXP), also contributes to the increased buildup of ROS in plant cells [80]. We observed that HS induced increased cellular H_2_O_2_ levels; however, NHCC leaves and roots, and *Arabidopsis* with *BcWRKY22* overexpression, exhibited the reduced accumulation of H_2_O_2_ (Figure 3c, Figure 4e, and Figure 5d). In *Arabidopsis*, a series of tiny proteins called CAT1, CAT2, and CAT3 are produced by the CAT gene. These proteins catalyze the clearing of hydrogen peroxide (H_2_O_2_) and are crucial for controlling ROS homeostasis. Specifically, under various environmental stresses, CAT2 is a crucial participant in H_2_O_2_ removal [81,82,83,84]. Interestingly, in NHCC leaves and roots overexpressing *BcWRKY22*, we observed an enhancement in CAT activity. Moreover, under HS conditions, the overexpressed tissues exhibited higher levels of CAT enzyme activity to effectively eliminate accumulated H_2_O_2_ than did the control (Figure 3f and Figure 5e). Meanwhile, the upregulation of *BcCAT2* gene expression, which governs CAT enzyme activity, was significantly enhanced in both the overexpressed leaves and the overexpressed roots (Figure 3g and Figure 5f). Although the regulation of CAT genes by other WRKY members in response to HS is unknown, our research demonstrates that BcWRKY22 directly stimulates the expression of BcCAT2 by binding to its promoter, suggesting a possible regulatory pathway involving HS-*BcWRKY22*-*BcCAT2*-CAT-H_2_O_2_.

## 5. Conclusions

In conclusion, our findings demonstrate that the activation of BcWRKY22, a member of the WRKY-IIe family, under heat stress conditions leads to the increased activity of the CAT enzyme through the upregulation of the expression of *BcCAT2*, a key regulator for CAT enzyme activity. This ultimately results in scavenging ROS being generated due to heat stress and enhancing the thermotolerance capacity of NHCC. The explanation of this mechanism can serve as a reference for the impact of antioxidant enzymes on plant heat tolerance, and also provide guidance for breeders in selecting heat-resistant crops, thereby addressing the issue of low yield in summer Chinese cabbage cultivation.

## Data Availability

Data will be made available upon request.

## Supplementary Materials

The following supporting information can be downloaded at: https://www.mdpi.com/article/10.3390/antiox12091710/s1. Figure S1: A phylogenetic tree constructed based on WRKYs of *Arabidopsis thaliana* and BcWRKY22; Figure S2: Identification of *BcWRKY22* overexpressed *Arabidopsis* DNA; Table S1: The primer list used in this article; Table S2: Analysis of the *BcCAT2* promoter elements by PlantCare.
