# Supplementary material for: BcWRKY22 Activates BcCAT2 to Enhance Catalase (CAT) Activity and Reduce Hydrogen Peroxide (H2O2) Accumulation, Promoting Thermotolerance in Non-Heading Chinese Cabbage (Brassica campestris ssp. chinensis)
Source: Antioxidants (Basel). 2023 Sep 1;12(9):1710. doi: 10.3390/antiox12091710 (PMC10525746; doi:10.3390/antiox12091710)
Supplement: Supplementary file 1 [file antioxidants-12-01710-s001.zip › Supplementary Figures.pdf]

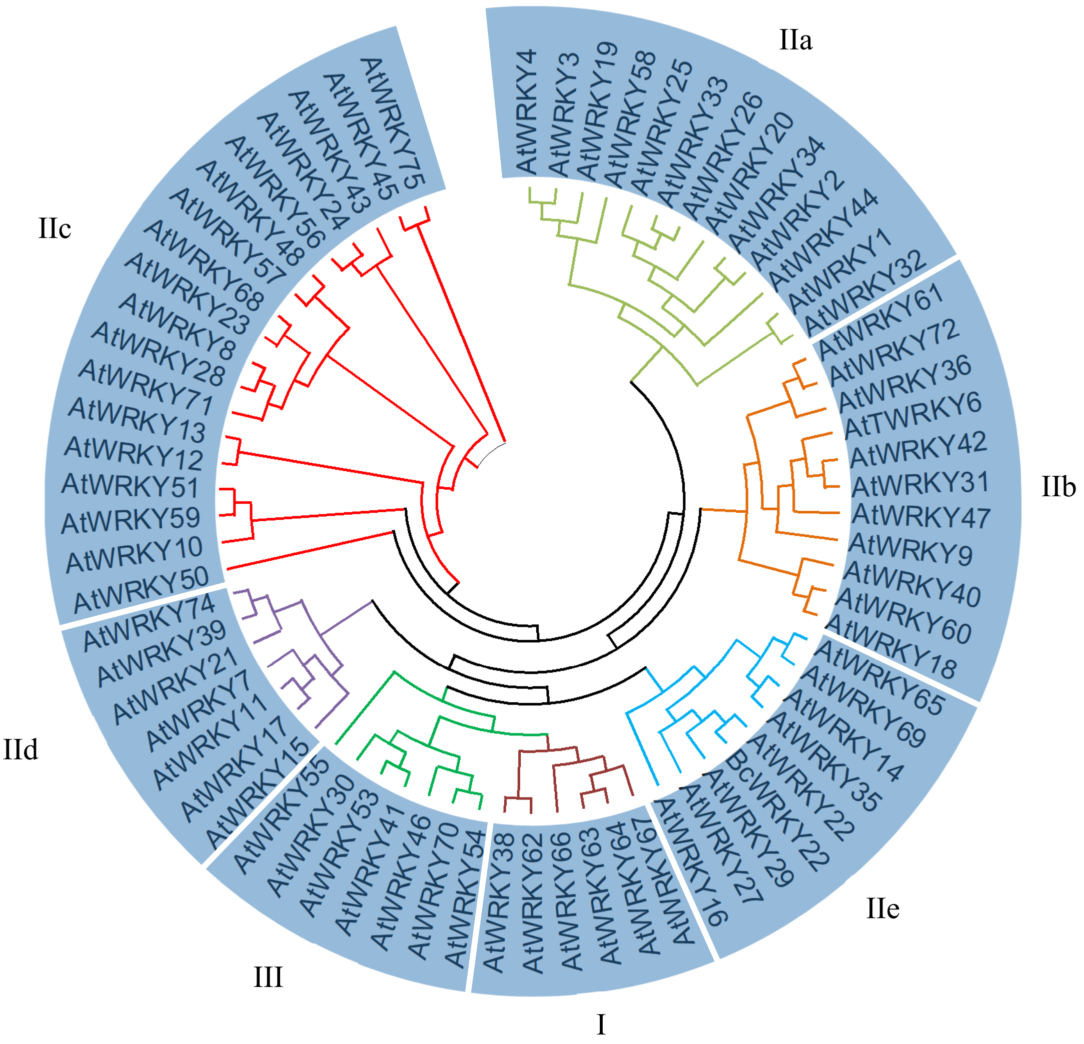

Figure S1. A phylogenetic tree constructed based on WRKYs of *Arabidopsis thaliana* and BcWRKY22.

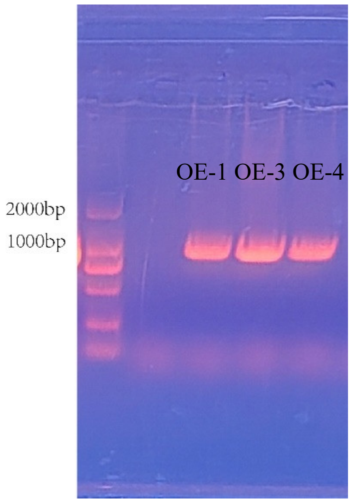

Figure S2. Identification of *BcWRKY22* overexpressed *Arabidopsis* DNA.
