# Supplementary material for: BcWRKY22 Activates BcCAT2 to Enhance Catalase (CAT) Activity and Reduce Hydrogen Peroxide (H2O2) Accumulation, Promoting Thermotolerance in Non-Heading Chinese Cabbage (Brassica campestris ssp. chinensis)
Source: Antioxidants (Basel). 2023 Sep 1;12(9):1710. doi: 10.3390/antiox12091710 (PMC10525746; doi:10.3390/antiox12091710)
Supplement: Supplementary file 1 [file antioxidants-12-01710-s001.zip › Supplementary Table S1.pdf]

Supplementary Table S1. The primers used in this article.	
Name	Sequences(5′ to 3′)
**Primers for gene cloning**	
BcWRKY22-F	ATGGCCGACGATTGGGATCTCCACGC
BcWRKY22-R	TCATATTCCACCGCTAGCTGTGGCAGCG
**Primers for isolation of promoter**	
P-cat-F	CAACCTACATTGTGATTCTCA
P-cat-R	AGTAGAAAGATAGAGAGAGTTTG
**Primers for gene qPCR**	
GAPC-F	AGAGCCGCTTCCTTCAACATCATT
GAPC-R	TGGGCACACGGAAGGACATACC
BcWRKY22-DL-F	CTAAGTCTCCCACGACCACG
BcWRKY22-DL-R	CCTCGAAGAAGTCCTCGCTC
DL-CAT-F1	ATCCAGGTCATTGATCCAGC
DL-CAT-R1	ATGATAGCAGGGCAAAAGGC
DL-BcDREB2A-F	TGATGTCCATGAGGGTGCAG
DL-BcDREB2A-R	TTCTCCTCCAGAAGCCCAGT
DL-BcHSFA2-F	GTTCGCGCTAATGAGCAAGG
DL-BcHSFA2-R	CGTCTCCATATCATCCTTCATTCT
DL-GolS1-F	GTCGCCTCCAGTTCACGG
DL-GolS1-R	GGGACGTCTGGTAGTATCGC
**Primers for subcellular localization**	
101- WRKY22-F	TCTTCACTGTTGATACATATGATGGCCGACGATTG
101- WRKY22-R	GCCCTTGCTCACCATGGATCCTATTCCACCGCTAG
**Primers for transient expression**	
101- WRKY22-F	TCTTCACTGTTGATACATATGATGGCCGACGATTG
101- WRKY22-R	GCCCTTGCTCACCATGGATCCTATTCCACCGCTAG
**Primers for expression in NHCC roots**	
2300- WRKY22-F	CGGGGGACTCTGCAGGTCGACATGGCCGACGATTG
2300- WRKY22-R	TGAACGATCTCTAGAGTCGACTATTCCACCGCTAG

**Primers for dual-luciferase vectors construction**	
Pgreen -cat-F	CTATAGGGCGAATTGGGTACCCATGAGAGTTTTGTT
Pgreen -cat-R	ATCGATACCGTCGACCTCGAGAGTAGAAAGATAGAG
**Primers for yeast one hybrid vectors construction**	
PjG4-22-F	GATTATGCCTCTCCCGAATTCATGGCCGACGATTG
PjG4-22-R	AGAAGTCCAAAGCTTCTCGAGTATTCCACCGCTAGC
Plac-CAT-1-f	TGAATTGAAAAGCTTTTAAGGTCAAGATTTTAAGGTCAAGATTT TAAGGTCAAGATTCTCGAGGCATGTGCT
Plac-CAT-1-r	AGCACATGCCTCGAGAATCTTGACCTTAAAATCTTGACCTTAAA ATCTTGACCTTAAAAGCTTTTCAATTCA
Plac-CAT-2-f	TGAATTGAAAAGCTTAACAAGTCAAAACTAACAAGTCAAAACTA ACAAGTCAAAACTCTCGAGGCATGTGCT
Plac-CAT-2-r	AGCACATGCCTCGAGAGTTTTGACTTGTTAGTTTTGACTTGTTAG TTTTGACTTGTTAAGCTTTTCAATTCA
**Primers for EMSA**	
MAL-22-F	GAGGGAAGGATTTCAGAATTCATGGCCGACGATTGG
MAL-22-R	CAGGTCGACTCTAGAGGATCCTCATATTCCACCGCTA
EMSA-CAT2-F	TATTCGTACAAGTACAACAAGTCAAAACTAATAAAATAAAATAGC
EMSA-CAT2-R	GCTATTTTATTTTATTAGTTTTGACTTGTTGTACTTGTACGAATA
EMSA-CAT2-COLD-F	TATTCGTACAAGTACAACAAGTCAAAACTAATAAAATAAAATAGC
EMSA-CAT2-M-F	TATTCGTACAAGTACAACAAAAAAAAACTAATAAAATAAAATAGC
EMSA-CAT2-M-R	GCTATTTTATTTTATTAGTTTTTTTTTGTTGTACTTGTACGAATA
