# Supplementary material for: BcWRKY22 Activates BcCAT2 to Enhance Catalase (CAT) Activity and Reduce Hydrogen Peroxide (H2O2) Accumulation, Promoting Thermotolerance in Non-Heading Chinese Cabbage (Brassica campestris ssp. chinensis)
Source: Antioxidants (Basel). 2023 Sep 1;12(9):1710. doi: 10.3390/antiox12091710 (PMC10525746; doi:10.3390/antiox12091710)
Supplement: Supplementary file 1 [file antioxidants-12-01710-s001.zip › Supplementary Table S2.pdf]

Table S2. Analysis of the *BcCAT2* promoter elements by PlantCare.

name	sequence	function
TGACG-motif	TGACG	cis-acting regulatory element involved in the MeJA-responsiveness
Unnamed__1	CGTGG	
chs-CMA1a	TTACTTAA	part of a light responsive element
Unnamed__4	CTCC	
Unnamed__4	CTCC	
Unnamed__4	CTCC	
Unnamed__4	CTCC	
Unnamed__4	CTCC	
Unnamed__4	CTCC	
Unnamed__4	CTCC	
Unnamed__4	CTCC	
Unnamed__4	CTCC	
Unnamed__4	CTCC	
Unnamed__4	CTCC	
Unnamed__4	CTCC	
Unnamed__4	CTCC	
Unnamed__4	CTCC	
Unnamed__4	CTCC	
Unnamed__4	CTCC	
MYB	TAACCA	
MYB	TAACCA	
MYB	TAACCA	
	motif_sequence	short_function
	motif_sequence	short_function
	motif_sequence	short_function
	motif_sequence	short_function
	motif_sequence	short_function
	motif_sequence	short_function
	motif_sequence	short_function
	motif_sequence	short_function
	motif_sequence	short_function
	motif_sequence	short_function
	motif_sequence	short_function
	motif_sequence	short_function
	motif_sequence	short_function
	motif_sequence	short_function
	motif_sequence	short_function
GATA-motif	AAGGATAAGG	part of a light responsive element
TCT-motif	TCTTAC	part of a light responsive element
O2-site	GATGATGTGG	cis-acting regulatory element involved in zein metabolism regulation
WRE3	CCACCT	

ERE	ATTTTAAA	
AAGAA-motif	GAAAGAA	
as-1	TGACG	
MYC	CATTTG	
MYC	CAATTG	
MYC	CAATTG	
TATA-box	ATATAA	core promoter element around -30 of transcription start
TATA-box	TATA	core promoter element around -30 of transcription start
TATA-box	ATATAT	core promoter element around -30 of transcription start
TATA-box	TATA	core promoter element around -30 of transcription start
TATA-box	TACAAAA	core promoter element around -30 of transcription start
TATA-box	TATAAAT	core promoter element around -30 of transcription start
TATA-box	TATAAA	core promoter element around -30 of transcription start
TATA-box	TATAA	core promoter element around -30 of transcription start
TATA-box	TATA	core promoter element around -30 of transcription start
Myb	CAACTG	
Myb	CAACTG	
Myb	TAACTG	
Myb	CAACTG	
W box	TTGACC	
TC-rich repeats	GTTTTCTTAC	cis-acting element involved in defense and stress responsiveness
CAAT-box	CCAAT	common cis-acting element in promoter and enhancer regions
CAAT-box	CAAT	
CAAT-box	CAAT	
CAAT-box	CAAAT	common cis-acting element in promoter and enhancer regions
CAAT-box	CAAT	
CAAT-box	CAAAT	common cis-acting element in promoter and enhancer regions
CAAT-box	CAAT	
CAAT-box	CAAT	
CAAT-box	CAAT	
CAAT-box	CAAT	
CAAT-box	CAAT	
CAAT-box	CAAT	
CAAT-box	CAAT	
CAAT-box	CCAAT	common cis-acting element in promoter and enhancer regions
CAAT-box	CAAT	
CAAT-box	CAAT	
CAAT-box	CAAT	
CAAT-box	CAAT	
CAAT-box	CAAT	
CAAT-box	CAAT	
CAAT-box	CAAT	
CAAT-box	CAAAT	common cis-acting element in promoter and enhancer regions

CAAT-box	CCAAT	common cis-acting element in promoter and enhancer regions
CAAT-box	CAAAT	common cis-acting element in promoter and enhancer regions
MYB-like sequence	TAACCA	
MYB-like sequence	TAACCA	
MYB-like sequence	TAACCA	
TCA	TCATCTTCAT	
DRE1	ACCGAGA	
CGTCA-motif	CGTCA	cis-acting regulatory element involved in the MeJA-responsiveness
TGA-element	AACGAC	auxin-responsive element
TGA-element	AACGAC	auxin-responsive element
STRE	AGGGG	
MBS	CAACTG	MYB binding site involved in drought-inducibility
MBS	CAACTG	MYB binding site involved in drought-inducibility
MBS	CAACTG	MYB binding site involved in drought-inducibility
